# New microhylid frog genus from Peninsular India with Southeast Asian affinity suggests multiple Cenozoic biotic exchanges between India and Eurasia

**DOI:** 10.1038/s41598-018-38133-x

**Published:** 2019-02-13

**Authors:** Sonali Garg, S. D. Biju

**Affiliations:** Systematics Lab, Department of Environmental Studies, University of Delhi, Delhi, 110 007 India

**Keywords:** Biodiversity, Biogeography, Phylogenetics, Taxonomy, Herpetology

## Abstract

Anurans in Peninsular India exhibit close biogeographical links with Gondwana as well as Laurasia, often explainable by the geological history of the Indian subcontinent; its breakup from Gondwanan landmasses followed by long isolation that resulted in diversification of endemic lineages, and subsequent land connections with Asia that enabled dispersal of widespread groups. Although widely distributed, the frog subfamily Microhylinae mostly comprises of geographically restricted genera found either in Southeast and East Asia or Peninsular India and Sri Lanka. Here we report a previously unknown microhylid from the Western Ghats in Peninsular India with closest relatives found over 2,000 km away in Southeast Asia. Based on integrated evidence from mitochondrial and nuclear DNA, adult and tadpole morphology, hand musculature, male advertisement call, and geographical distance, we recognize this enigmatic frog as a distinct new species and genus endemic to the Western Ghats. The discovery of *Mysticellus franki* gen. et sp. nov. and its close evolutionary relationship with the Southeast Asian genus *Micryletta* also provide insights on the biogeography of Microhylinae. Genus-level divergences within the subfamily suggest multiple Cenozoic biotic exchange events between India and Eurasia, particularly through postulated Eocene land bridges via Southeast Asia prior to accretion of the two landmasses.

## Introduction

The Western Ghats is a chain of mountains that stretches over 1,600 km in southwest India. It is part of a global biodiversity hotspot with remarkable amphibian diversity and endemism^1^. This region is also considered as a distinct biogeographical unit, a recognition chiefly garnered due to the role of various events in the geological history of the Indian subcontinent^2–13^. After the initial break-up of Madagascar-Seychelles-India from Africa and Australia-Antarctica blocks (∼140–130 Mya), followed by separation of the Indian subcontinent from Madagascar (∼88 Mya) and subsequently the Seychelles (∼65 Mya), the northward-drifting Indian plate is considered to have been a “biotic ferry”^14^ during its course to unite with the Eurasian plate (starting ∼55 Mya)^15,16^. The biotic elements that moved along with the erstwhile Indian landmass during the Late Cretaceous or Palaeogene experienced long periods of isolation resulting in the origin of several endemic anuran lineages in the Western Ghats^11,15^. The biogeographical links of ancient frog lineages such as Nasikabatrachidae have provided crucial evidence for tracing back the evolutionary history of present day amphibians in the Western Ghats^10^.

While the isolation of the Indian subcontinent is regarded as a unique event, the union of the Indian plate with Eurasia is an equally intriguing juncture that provided opportunity for faunal exchanges between these landmasses^4,17,18^. Despite the constantly changing landscape and climatic conditions that created physical barriers for the movement of anurans^19,20^, several recent radiations (such as dicroglossids, microhylids and rhacophorids) acquired wider distribution patterns in India as well as South and Southeast Asia. In this context, the Northeast Indian region is often considered as a ‘gateway’^21,22^ bridging the Indian subcontinent with the rest of Asia, particularly before the rise of the Himalaya and upliftment of the Tibetan plateau. These present day physical features are known to have played a significant role in determining the distribution patterns of anurans in South and Southeast Asia^23–25^. Currently, four biogeographical regions encompassing the Indian subcontinent—Himalaya, Indo-Burma, Sundaland and Western Ghats-Sri Lanka—are recognized as global biodiversity hotspots^26^.

The Narrow-mouthed anuran family Microhylidae is distributed throughout the tropics and currently comprises of 653 species in 53 genera and 13 subfamilies^27^. Although higher-level taxonomic placement of the recognized subfamilies and their phylogenetic relationships are still disputed^28–33^, based on the more widely used classification by Frost *et al*.^29^ as revised by Peloso *et al*.^33^, five microhylid subfamilies are found in Asia (Asterophryinae, Chaperininae, Kalophryninae, Melanobatrachinae and Microhylinae), of which only two are known to occur in the Indian peninsula (Melanobatrachinae and Microhylinae). Melanobatrachinae is monotypic with a single known species and restricted to the southern Western Ghats of India. On the other hand, Microhylinae is widely distributed in South, Southeast and East Asia. It is a large radiation comprising of 87 species in seven genera^27,34^. Phylogenetically, Microhylinae is sister to Dyscophinae, and the split between the two subfamilies has been a significant point of interest for biogeographical interpretations, largely due to the congruence of geological events such as the break-up of India–Seychelles–Madagascar landmass during the Late Cretaceous^12,13^ with the restricted geographical distribution of members of these subfamilies to Asia and Madagascar, respectively. Phylogenetic relationships among members of the subfamily Microhylinae have been discussed in several studies^12,29,31,33,35^. A few have also provided insights on origin, possible routes of colonization, and patterns of distribution, however, in larger contexts of either global or selected Asian microhylids^12,13,36,37^. Hence, a detailed phylogenetic investigation and divergence ages within the subfamily Microhylinae are still lacking.

Our recent explorations in Peninsular India have led us to the discovery of a previously unknown microhylid frog that could not be assigned to any of the known microhylid members of this region. Evidence from multiple approaches concordantly suggests the newly discovered microhylid to be a distinct species, which also warrants recognition of a new genus within the subfamily Microhylinae. This novel evolutionary lineage is endemic to the Western Ghats, with its closest known relatives found over 2,000 km (as the crow flies) in the Indo-Burma and Sundaland hotspots. Here, we provide a formal description of *Mysticellus franki* gen. et sp. nov. and discuss new phylogenetic insights highlighted through this finding, along with their biogeographical implications.

## Results

Amphibia Linnaeus, 1758

Anura Fischer von Waldheim, 1813

Microhylidae Günther, 1858 (1843)

Microhylinae Günther, 1858 (1843)

### *Mysticellus* gen. nov

**Figure 1:**
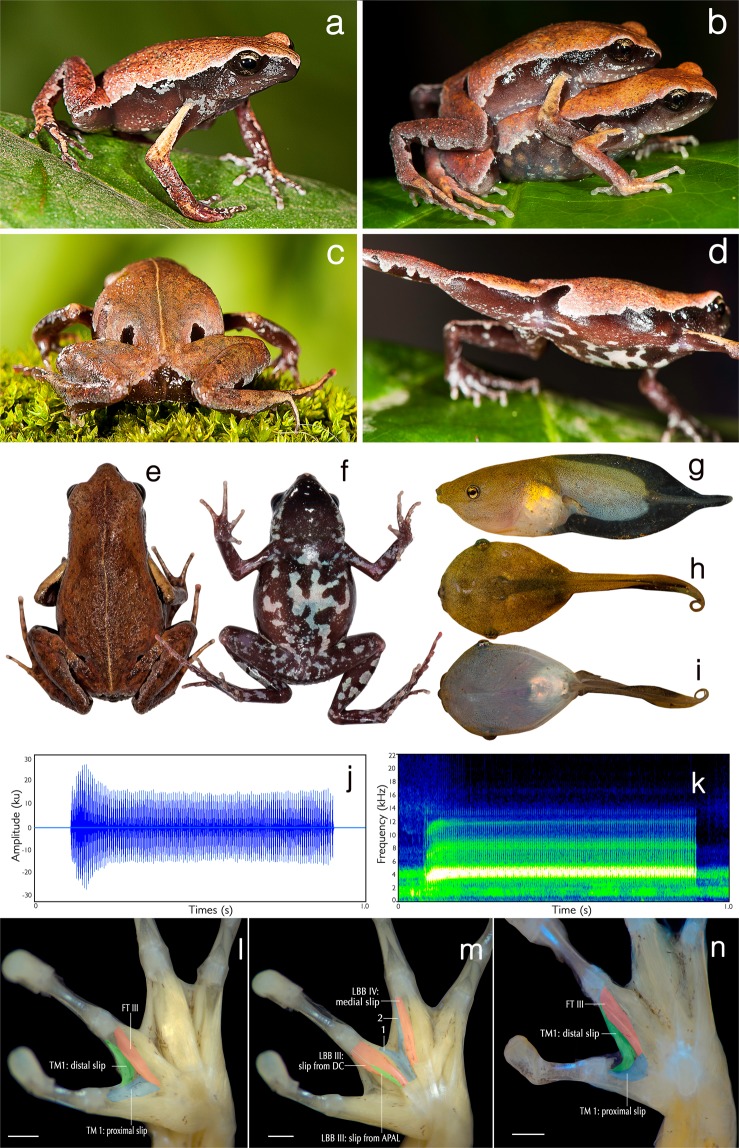
Diagnostic characteristics of *Mysticellus franki* gen. et sp. nov. (**a**–**f**) Adult in life. (**a**) Holotype (ZSI/WGRC/V/A/966, male) in dorsolateral view; (**b**) holotype (male) and paratype (ZSI/WGRC/V/A/971, female) in amplexus; (**c**) two ‘false-eye’ like spots on the back; (**d**) lateral markings; (**e**) dorsal view; (**f**) ventral view. (**g**–**i**) Tadpole in life. (**g**) Lateral view; (**h**) dorsal view; (**i**) ventral view. (**j**–**k**) Male advertisement call. (**j**) One second call segment showing pulsatile temporal structure; (**k**) spectrogram of one second call segment. (**l–n**) Hand musculature. (**l**–**m**) Palmar view of *Mysticellus franki* gen. et sp. nov. (SDBDU 2015.2870, left hand). (**l**) Flexor teres digiti III (FT III) passing ventrally to both slips of m. transversus metacarpus 1 (TM 1); (**m**) two previously unreported accessory flexor muscles on digiti III and IV (labeled as “1” and “2” respectively); (**n**) palmar view of *Micryletta inornata* (KU 328192, left hand) showing FT III dorsal to the proximal slip of TM 1 and ventral to the distal slip. Abbreviations: TM I: m. transversus metacarpus I, FT III: m. flexor teres digiti III, LBB III–IV: m. lumbricalis brevis digiti III–IV. Scale bars = 0.5 mm.

**Figure 2:**
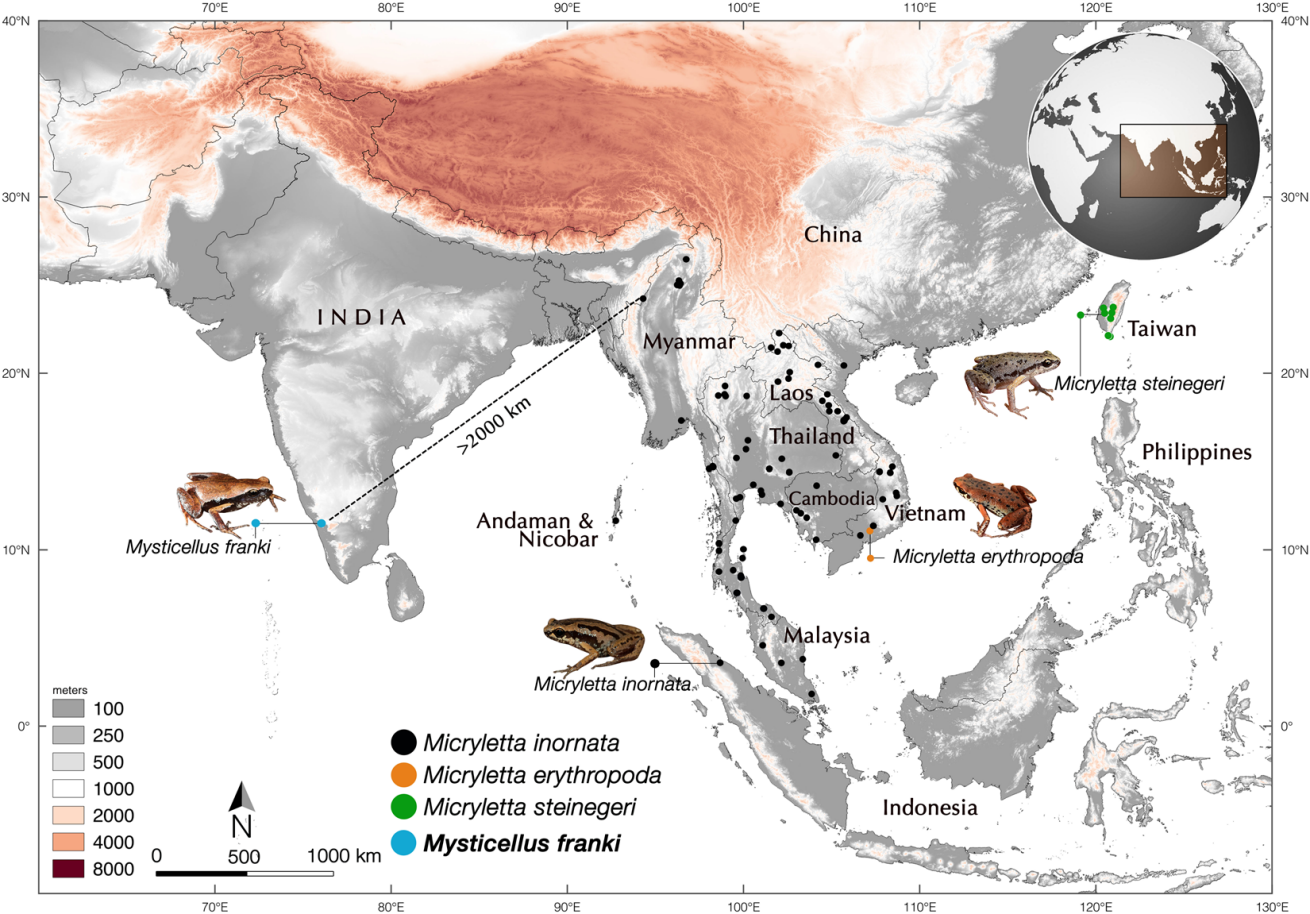
Distribution of *Mysticellus franki* gen. et sp. nov. and its closest generic relative *Micryletta*. Map prepared using QGIS 2.6.1 (http://www.qgis.org). Image credits: *Micryletta inornata* (M.A.M.M. Akil), *Micryletta erythropoda* (J. Rowley) and *Micryletta steinegeri* (N.A. Poyarkov Jr.).

**Figure 3:**
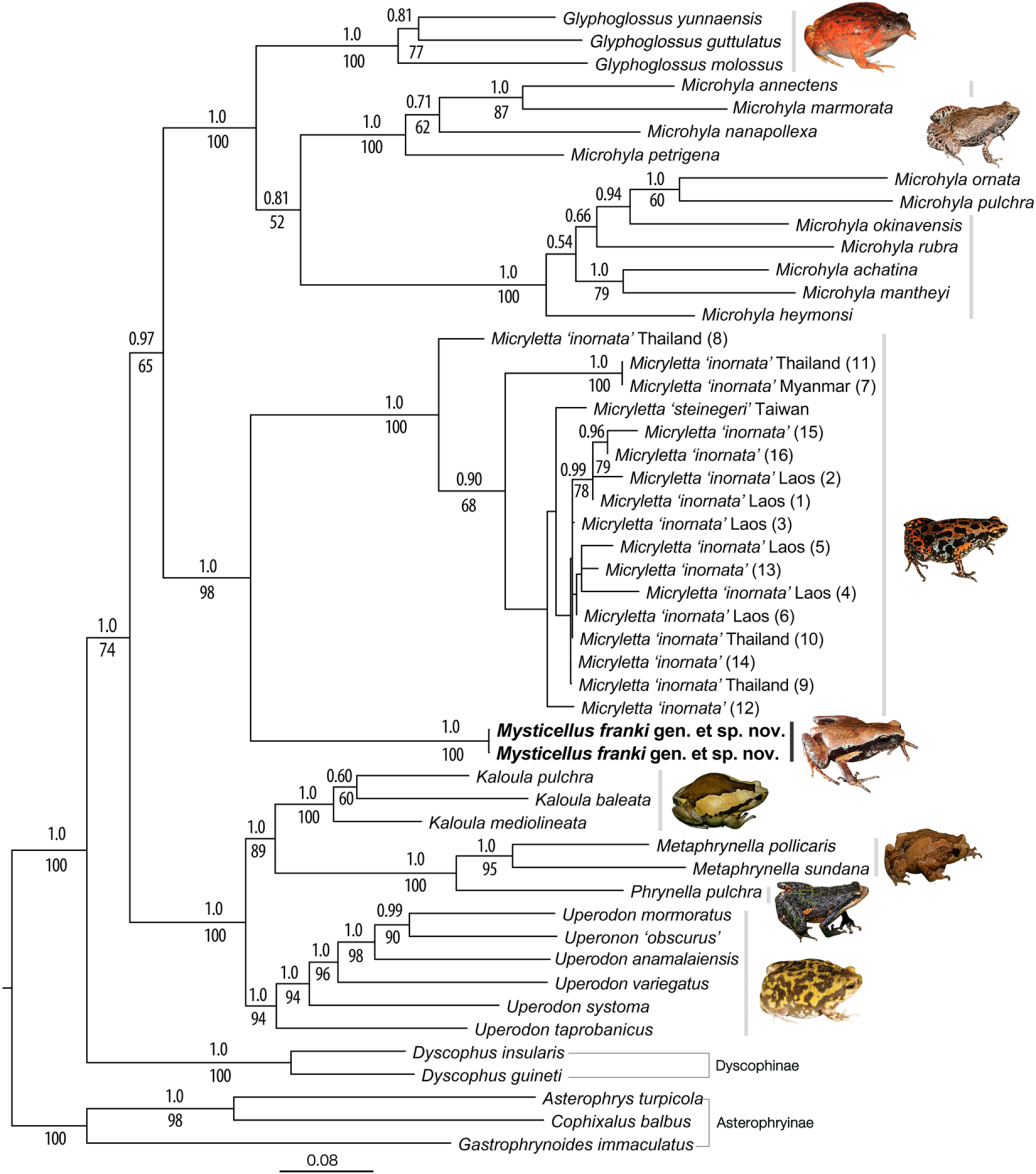
Maximum likelihood phylogram showing the phylogenetic position of *Mysticellus franki* gen. et sp. nov. in the subfamily Microhylinae. Bayesian Posterior Probabilities (BPP) and RAxML Bootstrap support values (BS) are shown above and below the branches, respectively. Image credits: *Glyphoglossus* (B. Tapley), *Metaphrynella* (A. Figueroa), *Micryletta* (N.A. Poyarkov Jr.) and *Phrynella* (D. Bickford).

**Figure 4:**
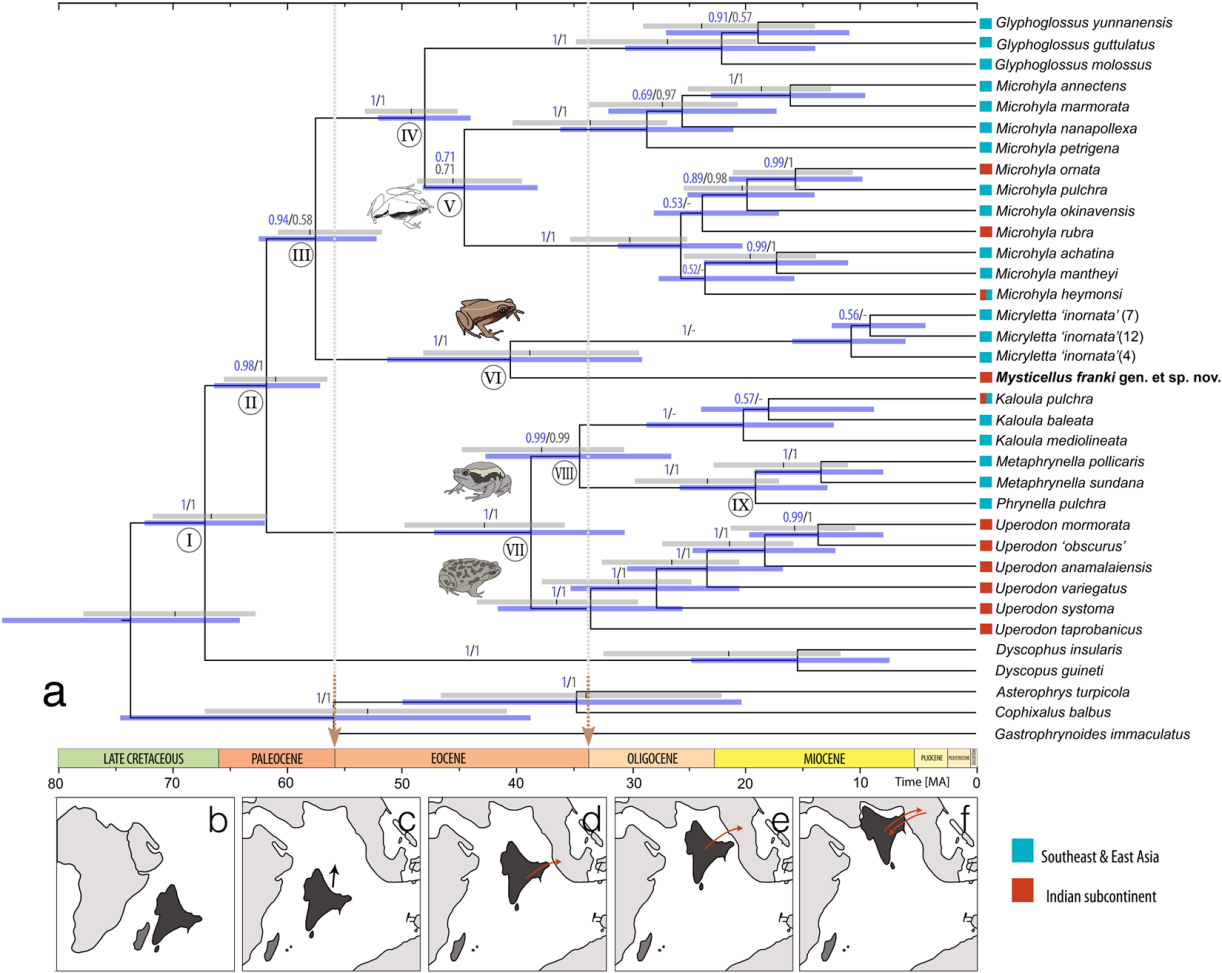
Estimated divergence ages in the subfamily Microhylinae and coinciding dispersal events. (**a**) Time-calibrated phylogeny from BEAST analysis using the Best-fit models of evolution. Node numbers are referenced in Table 1; node support values > 0.50 are provided above the branches (analysis A followed by B); 95% HPD intervals of the inferred age estimates are represented by blue (analysis A) and grey (analysis B) bars; geographical distribution of species are indicated alongside taxon labels. (**b**–**f**) Cenozoic position of the Indian subcontinent and postulated biotic exchange events between India and Eurasia. (**b**) Separated India and Madagascar landmasses (K/Pg boundary); (**c**) isolation of the northward drifting Indian subcontinent (Paleocene); (**d**) first Eocene land bridge between India and Southeast Asia through Sumatra; (**e**) second Eocene land bridge between India and mainland Southeast Asia through Myanmar-Malay Peninsula; (**f**) final accretion of India and Eurasia (Oligocene/Miocene). Suggestive paleomaps are based on Bossuyt and Milinkovitch^8^, Klaus *et al*.^52^ and Grismer *et al*.^53^.

urn:lsid:zoobank.org:act:6145F26C-E140-4BBF-9322-550047926EA2

(Figures 1–4; Supplementary Tables S1–S4).

### Type species

*Mysticellus franki* sp. nov.

### Etymology

The genus name, *Mysticellus*, is a masculine noun derived from the Latin *mysticus* (meaning mysterious) + *ellus* (a diminuitive), highlighting the ability of this small frog to remain out of sight despite its occurrence in wayside areas surrounding human settlements.

### Suggested common name

Mysterious Narrow-mouthed Frog.

### Diagnosis

The new genus *Mysticellus* differs from other Microhylinae genera by the combination of following characters: small adult snout-vent size (male SVL 23.0–27.5 mm, *N* = 5; female SVL 27.0–28.9 mm, *N* = 2), slender body; snout longer than eye length, male SL 2.8–3.0 mm, 2.9 ± 0.1 mm, *N* = 5, female SL 3.0–3.2 mm, 3.1 ± 0.1 mm, *N = *2 vs. male EL 2.4–2.6 mm, 2.5 ± 0.1 mm, *N* = 5; female EL 2.5–2.7 mm, 2.6 ± 0.1 mm, *N* = 2; absence of maxillary and vomerine teeth; finger and toe tips rounded, with small discs; presence of well-developed subarticular tubercles on all fingers and toes, rounded, alternating with additional smaller tubercles; prominent inner metatarsal tubercle and a small outer metatarsal tubercle on foot; webbing between fingers absent; rudimentary webbing between toes; lateral surfaces from tip of the snout up to the groin prominently dark blackish-brown; two prominent dark blackish-brown ‘false-eye’ like spots on either side of the groin extending just above the hind legs; a thin mid-dorsal line invariably extending from tip of the snout up to the vent; ventral surfaces of throat, belly, arms and legs dark brown with a violet tinge and various sized greyish-white blotches and speckles. This new genus can also be distinguished from other members of the subfamily by its hand musculature in having the m. flexor teres digiti III ventral to both slips of the m. transversus metacarpus 1^38^; and the dorsal surface with two previously unreported flexor muscles on digits III and IV, one lateral to the m. lumbricalis brevis digiti III, and the other medial to the medial slip of the m. lumbricalis brevis digiti IV (Fig. 1).

### Morphological comparison

The new genus *Mysticellus* differs from *Glyphoglossus* Günther, 1869 by its slender body (vs. robust), absence of maxillary and vomerine teeth (vs. present), horizontal pupil (vs. vertical), and well-developed tubercles on the hand (vs. not enlarged); differs from *Kaloula* Gray, 1831 by its smaller adult snout-vent size, SVL < 30 mm (vs. SVL > 30 mm), slender body (vs. robust), absence of ridge on posterior sides of choanae (vs. present), supratympanic fold absent (vs. present), finger and toe tips slightly enlarged (vs. enlarged with prominent discs), and inner metatarsal tubercle not enlarged (vs. enlarged and spatulate); differs from *Metaphrynella* Parker, 1934 by absence of ridge on posterior sides of choanae (vs. present), finger and toe tips slightly enlarged (vs. enlarged with prominent discs), webbing absent between fingers (vs. present), terrestrial habitat (vs. predominantly arboreal), and behavior of breeding around temporary water puddles (vs. tree holes); differs from *Microhyla* Tschudi, 1838 by its body relatively linear in shape (vs. triangular), lateral surfaces of the body prominently dark blackish-brown from tip of the snout up to the lower abdomen, tapering close to the groin and extending towards the dorsal surface just above the hind legs in the form of two prominent blackish-brown ‘false-eye’ like spots on either side (vs. dark colouration absent or discontinuous and ‘false-eye’ like spots absent), and prominent subarticular tubercles alternating with additional smaller tubercles (vs. absence of additional smaller tubercles); differs from *Micryletta* Dubois, 1987 by its tympanum externally indistinct (vs. distinct), and lateral surfaces of the body prominently dark blackish-brown from tip of the snout up to the lower abdomen, tapering close to the groin and extending towards the dorsal surface just above the hind legs in the form of two prominent blackish-brown ‘false-eye’ like spots on either side (vs. dark colouration absent or discontinuous and ‘false-eye’ like spots absent); differs from *Phrynella* Boulenger, 1887 by its finger and toe tips slightly enlarged (vs. enlarged with prominent discs), metatarsal tubercles separate (vs. united), and rudimentary webbing between toes (vs. nearly fully webbed); and differs from *Uperodon* Duméril and Bibron, 1841 by its slender body (vs. robust and globular), absence of ridge on posterior sides of choanae (vs. present), prominent subarticular tubercles alternating with additional smaller tubercles (vs. absence of additional smaller tubercles), lateral surfaces of the body prominently dark blackish-brown from tip of the snout up to the lower abdomen, tapering close to the groin and extending towards the dorsal surface just above the hind legs in the form of two prominent blackish-brown ‘false-eye’ like spots on either side (vs. absent), finger and toe tips slightly enlarged (vs. enlarged with prominent discs except in *U. systoma* and *U. globulosus*), inner metatarsal tubercle not enlarged (vs. enlarged or spatulate), and rudimentary webbing between toes (vs. prominent webbing, except in *U. systoma*).

### Contents

The new genus currently contains a single species *Mysticellus franki* sp. nov.

### *Mysticellus franki* sp. nov

urn:lsid:zoobank.org:act:B1FBD56B-B6B3-412A-8988-E527A082C4C5

(Figures 1–4; Supplementary Figs S1–S6; Supplementary Tables S1–S4).

### Etymology

The species name, *franki*, is a Latin genitive honoring evolutionary biologist Prof Franky Bossuyt (Vrije Universiteit Brussel), recognizing his role in global amphibian research and education, and particularly for his contribution to the study of Indian amphibians.

### Suggested common name

Franky’s Narrow-mouthed Frog.

### Holotype

ZSI/WGRC/V/A/966, an adult male, SVL 23.0 mm, from Suganthagiri (11°32′19″ N 76°3′14″ E, 852 m asl), Wayanad district, Kerala state, India, collected by Sonali Garg and SD Biju on 05 June 2015.

### Paratypes

ZSI/WGRC/V/A/967–970, four adult males, and ZSI/WGRC/V/A/971–972, two adult females, collected along with the holotype.

### Description of holotype *(measurements in mm)*

Small adult male (SVL 23.0), rather slender; head wider than long (HW 7.4, HL 6.3), flat above; snout nearly truncate in dorsal and ventral view, acute in lateral view, snout length (SL 2.9) longer than horizontal diameter of eye (EL 2.4); loreal region acute, rounded canthus rostralis; interorbital space flat, twice (IUE 3.2) as wide as upper eyelid (UEW 1.6) and internarial distance (IN 1.6); nostril oval, closer to snout (NS 0.7) than eye (EN 1.3); pupil oval; vomerine ridge absent; tongue small, not emarginated, spatulate, bearing no median lingual process. Arms long, forearm length (FAL 5.5) shorter than hand length (HAL 7.0); relative length of fingers I < IV < II < III (FL_I_ 2.5, FL_II_ 3.1, FL_III_ 4.8, FL_IV_ 2.7); finger tips rounded, slightly enlarged with small discs, fingers without dermal fringes, webbing absent between fingers; subarticular tubercles prominent, rounded, all present, alternating with additional smaller tubercles; three prominent palmar tubercles, middle tubercle rounded (PTL 0.5), outer tubercle oval (OPTL 0.7), inner tubercle rounded (IPTL 0.4); supernumerary tubercles present. Hind limbs long, thigh (THL 10.3) slightly shorter than shank (SHL 10.5) and shorter than foot (FOL 11.7); distance from the base of tarsus to the tip of toe IV (TFOL 17.0); toes long, relative length of toes I < V < II < III < IV; toe tips rounded, slightly enlarged with small discs, toes with weakly developed dermal fringes, rudimentary webbing between toes; subarticular tubercles prominent, oval, all present, alternating with additional smaller tubercles; inner metatarsal tubercle prominent (IMTL 0.8), oval; outer metatarsal tubercle small (OMTL 0.4), rounded; supernumerary tubercles present on toes (Fig. 1a–f; Supplementary Fig. S1).

Skin of snout and upper eyelids shagreened with scattered granular projections; anterior and posterior parts of back, and upper and lower parts of flank shagreened with more prominent granular projections compared to the snout region; dorsal surfaces of forelimb, thigh and shank shagreened with granular projections; skin surrounding the anal region prominently granular. Ventral surface of throat finely granular; chest, belly and limbs smooth with scattered granular projections (Fig. 1a–f; Supplementary Fig. S1).

### Colour of holotype in life

Dorsum brick red to reddish-brown; a thin mid-dorsal line extending from the tip of snout up to the vent; lateral surfaces prominently dark blackish-brown from tip of the snout up to the lower abdomen, tapering close to the groin and extending towards the dorsal surface just above the hind legs in the form of two prominent blackish-brown ‘false-eye’ like spots on either side; dorsal surface of forearm light brown and hand (including fingers) reddish-brown; dorsal surface of hind limbs (including toes) brick red to reddish-brown with faint greyish-brown transverse bands; posterior parts of thigh light brown, shank and tarsus reddish-brown. Ventral surfaces of throat, belly, fore- and hind limbs dark brown with a violet tinge and various sized greyish-white blotches and speckles (Fig. 1a–f).

### Diagnosis

As for *Mysticellus* gen. nov.

### Ecology and Behavior

A large number of animals usually aggregate around temporary water collection sites about two to three days after the first monsoon showers. Individuals were collected from grass adjacent to water puddles in a small wayside quarry (about 25 m^2^ area). The specific site was located close to a secondary forest. Breeding activities were observed only for four to five days, after which the animals disappeared and no individuals could be located despite repeated visits. Tadpoles (stage 34) were observed at the same site towards the end of July (Fig. 1g–i). Calling males exhibited a peculiar behavior of raising the hind part of their body displaying a pair of black ‘false-eye’ like spots. On some occasions, similar behavior was observed when individuals were disturbed, suggesting that the ‘false-eye’ spots may be serving a defensive role against predators (Fig. 1c).

### Vocalization

Males of *Mysticellus franki* sp. nov. produce a single type of call with pulsatile temporal structure. Calls have uniform intervals and are not delivered in groups. Characteristics of a single male call (ZSI/WGRC/V/A/966) are as follows: call duration, 1771.4 ms; call rise time, 91.4 ms; call fall time, 1681.9 ms; number of pulses, 141; pulse rate, 80.2 pulses per second; call spectrum with a single broad peak and mean dominant frequency of 3.7 kHz (Fig. 1j–k; Supplementary Fig. S5).

### Note

For colour description of holotype in preservation, secondary sexual characters, variations, hand musculature, and description of tadpole, see additional taxonomic description in the electronic supplementary material.

### Distribution

*Mysticellus franki* gen. et. sp. nov. is presently known only from the type locality in southern Western Ghats region of Kerala, Peninsular India (Fig. 2).

### Phylogenetic relationship

The ML and Bayesian phylogenies showed similar topologies and relationships were congruent with previous studies. Microhylinae was recovered as the sister group of Dyscophinae, with three members of Asterophryinae forming the outgroup clade. Within Microhylinae, all the known genera were recovered as well-supported clades (BPP > 0.95, BS > 80), except *Microhyla* for which monophyly was weakly supported (Fig. 3). Our phylogenetic analyses concordantly recovered *Mysticellus franki* gen. et sp. nov. (Western Ghats) as a distinct and well-supported sister lineage to genus *Micryletta* (Indo-Burma + Sundaland). The new lineage was also well differentiated in independent analyses of nuclear and mitochondrial gene datasets. Pairwise distances for the mitochondrial 16S rRNA gene sequences showed considerable genetic differentiation (minimum 8.2%) between the new species and members of its closest generic relative *Micryletta*. This was also concordant with genetic divergences observed among various known genera in the subfamily Microhylinae (Fig. 3; Supplementary Fig. S6; Supplementary Tables S1–S3). Hence, based on presented evidence, *Mysticellus* gen. nov. is phylogenetically defined as the most inclusive clade comprising *Mysticellus franki* gen. et sp. nov., but not members of the genus *Micryletta*.

### Divergence time

Based on dating estimations, *Mysticellus* gen. nov. diverged from its sister lineage *Micryletta* ~39.7 (40.6–38.8) Mya during the Eocene (Fig. 4; Table 1). These age estimates based on two different sets of substitution models were within the 95% confidence interval (CI) values derived from both the analyses. Considering the timings of continental drift during the Cenozoic, the split between these two genera is likely to have occurred as the Indian landmass established close proximity with mainland Southeast Asia through Myanmar-Malay Peninsula during Middle/Late Eocene (Fig. 4e). As evident from age estimates observed in subfamily Microhylinae, most genus-level clades were established during the Eocene, suggesting land bridge connections between India and Eurasia well before the final accretion of these landmasses (Fig. 4d–e). On the other hand, the Oligocene epoch largely witnessed diversification within genus-level endemic radiations, possibly due to isolation. Subsequently, upon India’s final collision and accretion with Eurasia during the Miocene, diversification and exchanges only at the species-level are likely to have been prevalent between India and the rest of Asia (Fig. 4f).

**Table 1:** Estimated divergence ages of major microhyline lineages.

Node	Divergence event	Estimated age ± SD (95% HPD confidence interval)		
		Best-fit models (Analysis A)	HKY model (Analysis B)	Average age
I	Split of Microhylinae from Dyscophinae	67.3 (62.1–72.6)	66.6 (61.6–71.6)	67.0 (K-Pg boundary)
II^	Spilt between (*Glyphoglossus* + *Microhyla* + *Micryletta* + *Mysticellus*) and (*Kaloula* + *Metaphrynella* + *Phrynella* + *Uperodon*)	61.9 (57.2–66.5)	61.0 (56.6–65.5)	61.5 (Paleocene)
III^	Spilt between (*Glyphoglossus* + *Microhyla*) and (*Micryletta* + *Mysticellus*)	57.6 (52.3–62.6)	58.0 (51.7–60.9)	57.8 (Paleocene/Eocene)
IV*	Spilt between *Glyphoglossus* and *Microhyla*	48.1 (44.1–52.2)	49.2 (45.1–53.2)	48.7 (Eocene)
V*	Spilt between two major *Microhyla* clades	44.6 (38.2–48.3)	45.5 (39.6–48.7)	45.1 (Eocene)
VI **	Split between *Micryletta* and *Mysticellus*	40.6 (29.1–51.4)	38.8 (29.5–48.1)	39.7 (Eocene)
VII**	Split between *Uperodon* and (*Kaloula* + *Metaphrynella* + *Phrynella*)	38.8 (30.6–47.3)	42.7 (35.8–49.8)	40.8 (Eocene/Oligocene)
VIII^	Split between *Kaloula* and (*Metaphrynella* + *Phrynella*)	34.6 (26.6–42.8)	37.8 (30.8–44.8)	36.2 (Eocene/Oligocene)
IX^	Split between *Metaphrynella* and *Phrynella*	19.2 (12.9–25.8)	23.3 (17.1–29.9)	21.3 (Oligocene/Miocene)

Ages are in Million years; HPD = highest posterior density. Best-fit models are provided in Supplementary Table S4. *Denotes first postulated Miocene exchange; **Denotes second postulated Miocene exchange; ^Denotes diversification events associated with periods of isolation.

## Discussion

### Phylogenetic and geographical relationship of the new genus

The discovery of a novel microhylid genus from the Western Ghats reemphasizes the importance of this region as a reservoir of several evolutionarily significant anuran lineages. It also indicates that extensive explorations can still result in identification of unknown taxa, which is necessary before a comprehensive understanding of existing diversity, their systematic relationships and patterns of biogeographical distribution can be achieved. The new frog *Mysticellus franki* gen. et sp. nov. is most closely related to the Southeast Asian genus *Micryletta*, which comprises of three recognized species and is largely endemic to Southeast Asia (namely Cambodia, Indonesia, Laos, Malaysia, Myanmar, Thailand, and Vietnam) and China, including Taiwan^27^. Even though molecular data have supported monophyly of *Micryletta*, the phylogenetic position of this clade has remained confusing^13,29,31–33,35–37^. In our phylogenetic analyses (Fig. 3), *Micryletta* shows a sister relationship with *Mysticellus* gen. nov. Together, these two genera form a clade that is sister to the group containing *Microhyla* + *Glyphoglossus*, which is largely consistent with the findings of Van der Meijden *et al*.^36^ and Peloso *et al*.^33^. Based on the current taxonomic arrangements in the family, we excluded *Chaperina* from our Microhylinae dataset^33^. However upon inclusion, *Chaperina* was found sister to the group containing *Microhyla* + *Glyphoglossus* in agreement with Kurabayashi *et al*.^13^ and Pyron and Wiens^32^, and the *Micryletta* + *Mysticellus* gen. nov. clade consistently occupied a basal position to these groups. Previous studies have also reported the presence of genus *Micryletta* in Andaman and Nicobar islands and Manipur in Northeast India^39,40^. However, based on close geographical proximity, the populations from Andaman and Nicobar islands are likely to be similar to *Micryletta inornata* originally described from the Indonesian island of Sumatra, whereas those from Manipur could be related to members in the adjacent regions of mainland Southeast Asia. Further molecular and morphological confirmation will be necessary to ascertain the identity of these records. Yet, despite the ambiguity in species-level identification of several currently known *Micryletta* populations, their distribution pattern suggests that the genus is largely restricted to the Indo-Burma and Sundaland biodiversity hotspots, with its range extending up to East Asia. On the other hand, its sister group *Mysticellus franki* gen. et sp. nov. is a distinct lineage found in Peninsular India.

### Colonization of subfamily Microhylinae

The Southeast Asian affinity of the new frog also provides interesting biogeographical insights. The family Microhylidae originated on Gondwanaland and acquired wide distribution on most continents including Africa, Asia, Australia, and North and South America^12,23,36,41^. Colonization and diversification of Microhylidae remains a complex subject that requires understanding of all possible dispersal routes, vicariance events, as well as major geological and climatological changes ever since the Early Cretaceous, as various landmasses of Gondwanan origin drifted towards their present day continental positions^12,13,36^. The subfamily Microhylinae, however, originated during the Late Cretaceous and is restricted to South, Southeast and East Asia^8,12,13,17,36^. Ancestors of Microhylinae (Asia) split from Dyscophinae (Madagascar), an event that coincides with the separation between Madagascar and Indian subcontinent, subsequent to which the latter moved northwards (Fig. 4b,c)^8,12,36^. The long isolation of the Indian subcontinent during the Late Cretaceous (Fig. 4c) led to the origin and diversification of several microhylid lineages, many of which probably remained restricted to the southern peninsular region by the Deccan traps that erupted around the Cretaceous-Palaeogene (K/Pg) boundary^8,11,12^, and later dispersed to Asia through various terrestrial connections.

In general, the dispersal route that formed following the collision of India with Asia has often been considered instrumental in faunal exchange between these landmasses^4,5,7,9,42–45^, particularly for colonization of anuran groups of Late Cretaceous Gondwanan origin from India to Asia^8,11–13,25^. Although the same may be regarded as the most parsimonious explanation for colonization of Microhylinae into Asia, a single dispersal event is less likely to have resulted in the origin of multiple microhyline lineages with Indo-Southeast Asian affinity since the peninsular elements remained isolated by the massive Deccan traps for a long duration after the K/Pg boundary^8,11^. If microhylids are believed to have been more widespread on the Indian subcontinent prior to the Deccan Traps, especially in the northern regions, even then their colonization solely through the Himalaya-Tibetan Plateau-Northeast Indian route would have been difficult during Oligocene–Miocene because of the enormous geological processes (subduction vs. orogeny), topographical changes (connection vs. isolation) and climatic fluctuations (favorable vs. unfavorable) that resulted after India’s collision^4,6,8,19,24,25,46,47^. These massive changes had important consequences to the movement of amphibians due to their limited dispersal abilities^24,48^.

In an alternate scenario, as the Indian subcontinent drifted closer towards Asia during the Palaeogene, it is postulated to have made brief land connections with insular Southeast Asia and mainland Southeast Asia, opening dispersal opportunities early during the Eocene^49–51^. Our discovery of a new microhylid genus from the Western Ghats with closest links in Southeast Asia provides evidence for at least one or more dispersal events between India and Southeast Asia (Figs 3 and 4), well before the Tibetan-Himalayan route became available (Fig. 4d–f). Most microhylines are restricted either to Peninsular India and Sri Lanka, or Indo-Burma and Sundaland, suggestive of early land connections between these regions followed by isolation, as India drifted closer towards Eurasia. The most recent common ancestor (MRCA) of *Glyphoglossus* + *Microhyla* (~48.7 Mya) and the MRCA of two large radiations of *Microhyla* seem to have utilized Early Eocene land bridges (~45.1 Mya) to colonize Southeast Asia through direct land connection with Sundaic regions such as Sumatra (believed to have been a single landmass). Whereas, MRCAs of microhyline radiations such as *Uperodon* + (*Kaloula* + *Metaphrynella* + *Phrynella*) (~40.8 Mya) and *Micryletta* + *Mysticellus* (~39.7 Mya) possibly dispersed through a second land bridge with Myanmar–Malay Peninsula later during the Eocene^25,52,53^ (Fig. 4; Table 1). Since the exact path and timing of landmass movement remains debatable^49,51,54^, such connections could have either been multiple events intermitted with brief isolation periods^53^, or the Indian subcontinent could have followed a counter clockwise trajectory along the western boundary of Southeast Asia (Sumatra–Myanmar–Malay Peninsula) forming more prolonged connections [e.g., ref.^52^ and references therein]. The subsequent period starting around Oligocene is largely represented by diversification within the genus-level endemic radiations (Fig. 4), suggesting periods of geographical isolation apart from the continued separation of Indian Peninsula by the Deccan traps, until India’s hard collision with Asia around Miocene. Several widespread microhylid species, particularly those with distributions north of the Deccan traps (such as *Microhyla* spp), could have then utilized the newly created dispersal opportunities to move into Asia. At the same time, Southeast Asian taxa in groups such as *Microhyla, Micryletta* and *Kaloula* probably also recolonized parts of Northeast India through these connections (Fig. 4). Such exchanges between India and Southeast Asia would have continued after the closure of Tethys sea, until the upliftment of Himalaya and Tibetan plateau, and embayment in Assam and Myanmar regions during the Miocene^8^. The latter geotectonic features once again restricted faunal exchange between the Indian subcontinent and neighboring regions^24,46^, probably accounting for the present day species-level endemism patterns observed in several Indo-Asian microhyline groups.

### Multiple faunal exchanges between India and Asia

Phylogenetic evidence from the study of Microhylinae is suggestive of multiple dispersal events between India and Asia through postulated land bridges right from Eocene up to Miocene. While the more widely discussed single Miocene exchange after India’s final accretion with Asia explains the affinities between Indian and Southeast Asian faunal elements, it does not provide a convincing justification for early genus-level divergences and the parallel occurrence of endemism as well as widespread distribution patterns observed in Microhylinae. Hence, a trichotomy of (1) Eocene land bridges between India and Southeast Asia prior to the final Indo-Asia accretion, also termed as the “Eocene exchange hypothesis”^53^, (2) prolonged isolation of Indian Peninsula by the Deccan traps (since the K/Pg boundary) along with intermitted periods of isolation during Eocene–Oligocene, and (3) limited Miocene exchange between northern Indian regions (presently comprising of Himalaya–Tibetan plateau and Northeast India) and Asia due to complex geological and paleoclimatological events associated with Indo-Asia collision—explains early colonization of certain faunal groups from India to Southeast Asia, as well as the observed patterns of regional endemism and widespread distributions. Future research would benefit from wider taxon sampling across faunal groups with Indo-Southeast Asian affinities, and a better understanding of the exact timings and positions of land bridges, which remain debatable till date.

## Methods

### Field surveys and sampling

Field surveys were conducted during the monsoon season in the months of June and July (2015). Sampled individuals were photographed and euthanized in aqueous solution of Tricaine methanesulfonate (MS-222). Adults were fixed in 4% formalin and preserved in 70% alcohol. Tadpoles were preserved in 10% neutral-buffered formalin. Prior to preservation, tissue samples from the tadpole (tail muscle) and adults (thigh muscle) were taken in absolute alcohol for molecular studies and stored at −20 degree in Systematics Lab, Department of Environmental Studies, University of Delhi (SDBDU). Male advertisement calls were recorded using a Marantz PMD620 solid-state digital recorder (44.1 kHz sampling rate, 16-bit resolution) with a handheld Sennheiser ME 66 unidirectional microphone. Air temperature (dry bulb and wet bulb) at the calling site was recorded to the nearest 0.1 °C. Type specimens are deposited in Zoological Survey of India–Western Ghats Regional Centre (ZSI-WGRC), Kozhikode and referred specimens are available in the SDBDU collection. The study was conducted with permission and following the guidelines by the concerned authorities in the State Forest Department, Government of Kerala (Permit No. WL10-25421/2014).

### DNA extraction, PCR and sequencing

Total genomic DNA was extracted from adult and tadpole tissue samples using Qiagen DNeasy tissue kit, following the manufacturer’s protocols. Based on availability of GenBank data, the following gene fragments were PCR-amplified using previously published primers: two mitochondrial (mt) genes—ribosomal subunit 16 S rRNA (16 S, ∼560 bp, primer set 16Sar and 16Sbr)^55^ and cytochrome oxidase I (CO1, ∼650 bp, primer set Chmf4 and Chmr4)^56^, and four nuclear (nu) genes—brain-derived neurotrophic factor (BDNF, ∼700 bp, primer set BDNF.Amp.F1 and BDNF.Amp.R1)^36^, histone H3 (His3, ∼330 bp, primer set H3F and H3R)^57^, seven in absentia homolog 1 (SIA1, ∼399 bp, primer set SIA1 and SIA2)^58^ and tyrosinase (Tyr, ∼530 bp, primer set TyrC and TyrG)^15^. Sequencing was performed on both strands using the BigDye terminator cycle sequencing kit on ABI 3730 automated DNA sequencer (Applied Biosystems). Nucleotide sequences were checked and assembled in ChromasPro v1.34 (Technelysium Pty Ltd.), and deposited in the Genbank under accession numbers MK285340–MK285351.

### Phylogenetic analyses

DNA sequences for 43 taxa representing all the recognized genera in subfamily Microhylinae and five outgroup taxa (Asterophryinae and Dyscophinae) were retrieved from the GenBank (Supplementary Table S1). In addition, newly generated sequences of the unknown microhylid were included in the study. Sequences were aligned using MEGA 6.0^59^. For non-coding DNA, ambiguous portions were identified manually and excluded from the analyses. A combined mitochondrial and nuclear DNA dataset of 3,088 basepairs was assembled and phylogenetic estimations were made based on Maximum Likelihood (ML) and Bayesian analyses. The data matrix was partitioned by genes and appropriate likelihood models were estimated independently using Modeltest 3.4^60^ that yielded GTR + G + I as the best-fitting model for each gene. We used RAxML 7.3.0^61^ as implemented in raxmlGUI 1.1^62^ to perform ML searches with GTRGAMMA model recommended in RAxML^61^. The rapid bootstrap algorithm^63^ was executed with 1000 replicates and a thorough ML search based on 200 independent runs (20 percent of bootstrap replicates). Bootstrap support (BS) was computed on the resulting majority-rule consensus tree. Bayesian analysis was performed in MrBayes 3.1.2^64^ using GTR + G + I model across all gene partitions. Two parallel runs of four Markov Chain Monte Carlo (MCMC) chains were executed for 10 million generations with sampling at every 1000th generation. Convergence of the parallel runs was determined by split frequency of <0.01 standard deviations and potential scale reduction factors of ∼1.0. Stationarity of the likelihood scores and effective sample sizes (ESS) for all parameters were checked in Tracer v1.6^65^. Trees were summarized with a burn-in value of 10 percent and Bayesian Posterior Probabilities (BPP) were used to estimate clade credibility. Additionally, ML trees were generated for each nuclear and mitochondrial gene in order to independently assess distinctness of the new lineage. Uncorrected intra- and interspecific pairwise genetic distances were computed for the 16S mitochondrial gene sequences using PAUP* 4.0b10^66^. Pairwise identity was graphically visualized using the Sequence Demarcation Tool v1.2^67^.

### Dating estimates

For estimation of divergence times in the subfamily Microhylinae, an uncorrelated lognormal relaxed-clock analysis was performed in the software package BEAST v2.4.4^68^. The data set was partitioned by genes and best-fitting substitution models (Supplementary Table S4) were determined in PartitionFinder 2.1.1^69^ using the ‘greedy’ search option and ‘mrbayes’ models under Bayesian information criterion. The Birth-death model was used for tree priors as it takes into account two parameters, i.e. lineage birth as well as extinction, while estimating phylogenetic diversification^70^. All other parameters were left as default. For dating of nodes, three calibration points were used following Van Bocxlaer *et al*.^12^ with normally distributed priors: (1) the split between Dyscophinae and Microhylinae representing the most recent common ancestor (MRCA) of Microhylinae was calibrated at a mean age of 68 Mya and standard deviation of 3.5 Mya, (2) the MRCA of *Kaloula* and *Microhyla* was calibrated at a mean age of 62 Mya and standard deviation of 3.5 Mya, and (3) the MRCA of *Glyphoglossus* and *Microhyla* was calibrated at a mean age of 48 Mya and standard deviation of 2.5 Mya. Four independent runs with MCMC chain length of 50 million each were executed in BEAST v2.4.4^68^ and trees were sampled at every 500th generation. Convergence of the runs was assessed in Tracer v1.6^65^ and the initial 10 percent trees were discarded as burn-in. The results were combined in LogCombiner^68^ and ESS values of over 200 were largely achieved for all parameters, except posterior (ESS = 126). In order to avoid overparameterization, an additional analysis of 100 million generations with 10 percent burn-in was performed using only the HKY model for each locus, which yielded ESS values over 1000 for all parameters.

### Adult morphology

Sex and maturity of specimens were determined either by the presence of secondary sexual characters (nuptial pads and vocal sacs in males) or by examining the gonads through a small ventral incision. Only adult specimens were used for morphometric studies. Measurements were taken to the nearest 0.1 mm by using a digital slide-caliper or a binocular microscope with a micrometer ocular. Measurements and associated terminologies follow Biju *et al*.^71,72^; for abbreviations see electronic supplementary material. All measurements provided in the taxonomy section are in millimeters. Webbing formulae and the degree of webbing is described following Biju *et al*.^73^. Measurements and photographs were taken for the right side of the specimens, except when a character was damaged, in which case they were taken for the left side.

### Tadpole morphology

The tadpoles were morphologically examined and staged according to Gosner^74^. Descriptions are based on stage 34 tadpoles (*N* = 3). Measurements were taken to the nearest 0.01 mm using a stereomicroscope with a micrometer ocular or a digital slide-caliper. Measurements and larval terminologies were adapted from McDiarmid and Altig^75^. Oral morphology and margins of papillae were visualized using blue ink.

### Hand musculature

Palmar musculature was examined under stereomicroscope after dissection and removal of superficial layers (see electronic supplementary material). Dissection procedures, character sampling, terminologies and abbreviations follow Burton^38,76^ and Blotto *et al*.^77^.

### Acoustic analysis

Temporal and spectral properties were measured for a single call using Raven Pro 1.4^78^. Dominant frequency was measured after averaging spectrum over the entire call. Terminologies and graphical representation of the analysed call properties follow Bee *et al*.^79^.

### Nomenclatural acts

This article is published in an electronic journal with an ISSN (2045–2322), and has been archived in PubMed Central. Taxonomic nomenclature published in this article conforms to the requirements of the amended International Code of Zoological Nomenclature (ICZN), and hence is available under ICZN. This publication and the nomenclatural acts it contains have been registered in ZooBank, the proposed online registration system for the ICZN. The ZooBank LSID (Life Science Identifier) for this publication can be resolved and the associated information viewed through any standard web browser by appending the LSID to the prefix ‘http://zoobank.org/’. The LSID for this publication is urn:lsid:zoobank.org:pub:522D72B4-87B3-4A9B-B533-EA794695755D; LSID for *Mysticellus* gen. nov. is urn:lsid:zoobank.org:act:6145F26C-E140-4BBF-9322-550047926EA2; LSID for *Mysticellus franki* sp. nov. is urn:lsid:zoobank.org:act:B1FBD56B-B6B3-412A-8988-E527A082C4C5.

## Supplementary information

Electronic Supplementary Material
