# Author Correction: New microhylid frog genus from Peninsular India with Southeast Asian affinity suggests multiple Cenozoic biotic exchanges between India and Eurasia

**DOI:** 10.1038/s41598-019-48631-1

**Published:** 2019-08-28

**Authors:** Sonali Garg, S. D. Biju

**Affiliations:** Systematics Lab, Department of Environmental Studies, University of Delhi, Delhi, 110 007 India

Correction to: *Scientific Reports* 10.1038/s41598-018-38133-x, published online 13 February 2019

In this Article, the legend of Table 1 is incorrect:

“Ages are in Million years; HPD = highest posterior density. Best-fit models are provided in Supplementary Table S4. *Denotes first postulated Miocene exchange; **Denotes second postulated Miocene exchange; ^Denotes diversification events associated with periods of isolation.”

should read:

“Ages are in Million years; HPD = highest posterior density. Best-fit models are provided in Supplementary Table S4. *Denotes first postulated Eocene exchange; **Denotes second postulated Eocene exchange; ^Denotes diversification events associated with periods of isolation.”

