# Supplementary material for: New microhylid frog genus from Peninsular India with Southeast Asian affinity suggests multiple Cenozoic biotic exchanges between India and Eurasia
Source: Sci Rep. 2019 Feb 13;9:1906. doi: 10.1038/s41598-018-38133-x (PMC6374391; doi:10.1038/s41598-018-38133-x)

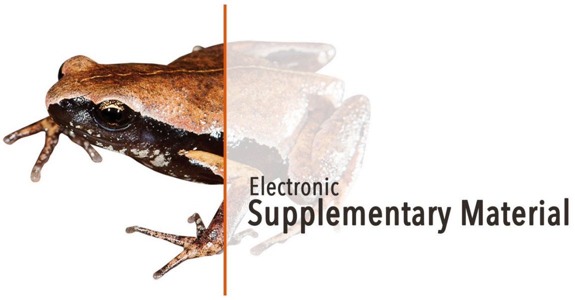

New microhylid frog genus from Peninsular India with Southeast Asian affinity suggests multiple Cenozoic biotic exchanges between India and Eurasia

Sonali Garg[1] & SD Biju[1]*
[1]Systematics Lab, Department of Environmental Studies
University of Delhi, Delhi 110 007, India
*Corresponding author | sdbiju@es.du.ac.in

Additional taxonomic description of *Mysticellus franki* gen. et sp. nov.

Colour of holotype in preservation. Dorsum greyish-brown, upper eyelids and center of the dorsum slightly darker greyish-brown, lateral surfaces prominently brownish-black from tip of the snout up to the lower abdomen, tapering close to the groin and extending towards the dorsal surface just above the hind legs in the form of two prominent brownish-black ‘false-eye’ like spots on either side; dorsal surface of forearm light brown, hand (including fingers) dark greyish-brown; dorsal surface of hind limbs (including toes) greyish-brown with faint dark brown transverse bands. Ventral surface of throat dark greyish-brown, chest, belly and limbs brown to dark brown with various sized off-white blotches and speckles, forearm and thighs light brown (Supplementary Fig. S1).

Secondary sexual characters. *Male:* nuptial pad on finger I absent; distinct calling patch on the throat (Fig. 1f; Supplementary Fig. S1). *Female* (ZSI/WGRC/V/A/971): over 150 eggs, pigmented (diameter 1.1 ± 0.3 mm, *N* = 20), large mass of transparent jelly above and below the egg mass.

Variations. Colour and markings are almost similar in all the type specimens (Supplementary Figs S2–S4). SDBDU 2015.2863: dorsal colour dark brick red in life and dark greyish-brown in preservation (Supplementary Fig. S3c). Measurements for the type series (range, mean and standard deviation, respectively), males (*N* = 5) followed by females (*N* = 2), are as follows: SVL 23.0 – 27.5, 24.4 ± 1.8 (m), 27.0 – 28.9, 28.0 ± 1.3 (f); HW 7.4 – 8.2, 7.6 ± 0.3 (m), 8.0 – 8.7, 8.4 ± 0.5 (f); HL 6.4 – 7.7, 6.8 ± 0.5 (m), 7.1 – 7.8, 7.5 ± 0.5 (f); IFE 3.2 – 3.9, 3.6 ± 0.3 (m), 3.5 – 3.8, 3.7 ± 0.2 (f); IBE 6.5 – 7.4, 6.9 ± 0.3 (m), 7.2 – 7.4, 7.3 ± 0.1 (f); SL 2.8 – 3.0, 2.9 ± 0.1 (m), 3.0 – 3.2, 3.1 ± 0.1 (f); IUE 3.1 – 3.5, 3.2 ± 0.2 (m), 3.1 – 3.4, 3.3 ± 0.2 (f); UEW 1.6 – 1.8, 1.6 ± 0.1 (m), 1.8 – 1.9, 1.9 ± 0.1 (f); IN 1.6 – 1.7, 1.7 ± 0.1 (m), 1.8 – 1.9, 1.9 ± 0.1 (f); MN 5.6 – 6.2, 5.7 ± 0.3 (m), 6.0 – 6.3, 6.2 ± 0.2 (f); EN 1.3 – 1.6, 1.4 ± 0.1 (m), 1.4 – 1.4, 1.4 ± 0.0 (f); NS 0.7 – 0.9, 0.8 ± 0.1 (m), 1.0 – 1.0, 1.0 ± 0.0 (f);

EL 2.4 – 2.6, 2.5 ± 0.1 (m), 2.5 – 2.7, 2.6 ± 0.1 (f); MN 2.4 – 2.6, 2.5 ± 0.1 (m), 2.5 – 2.7, 2.6 ± 0.1 (f); FAL 5.5 – 6.8, 6.0 ± 0.5 (m), 6.2 – 6.9, 6.6 ± 0.5 (f); HAL 7.0 – 7.5, 7.1 ± 0.2 (m), 7.2 – 7.7, 7.5 ± 0.4 (f); THL 10.3 – 11.3, 10.7 ± 0.4 (m), 11.6 – 12.3, 12.0 ± 0.5 (f); SHL 10.5 – 11.7, 10.9 ± 0.5 (m), 11.8 – 12.4, 12.1 ± 0.4 (f); FOL 11.6 – 12.2, 11.9 ± 0.3 (m), 12.5 – 13.3, 12.9 ± 0.6 (f); TAFOL 17.0 – 18.6, 17.6 ± 0.6 (m), 18.5 – 19.3, 18.9 ± 0.6 (f); OMTL 0.4 – 0.5, 0.5± 0.0 (m), 0.5 – 0.5, 0.5 ± 0.0 (f); IMTL 0.8 – 1.0, 0.9 ± 0.1 (m), 0.8 – 0.9, 0.9 ± 0.1 (f).

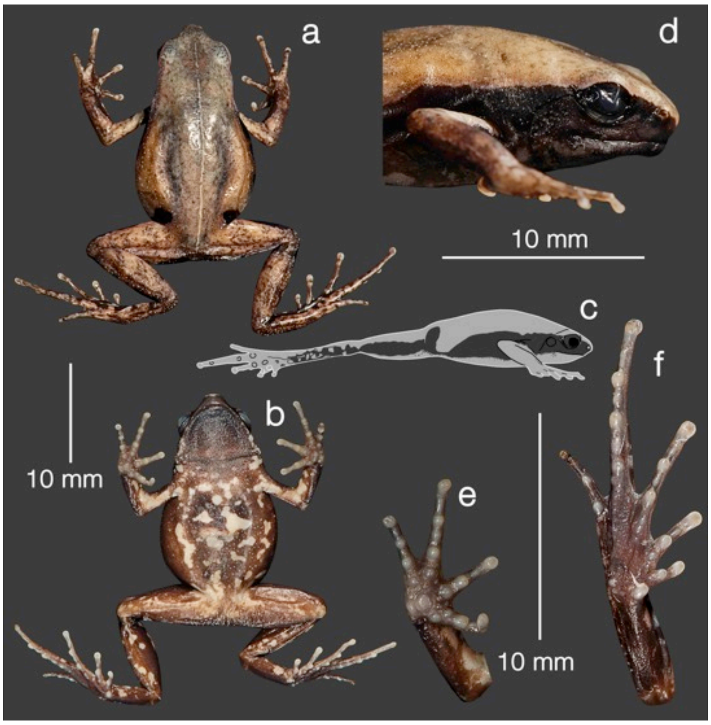

Supplementary Figure S1. Holotype specimen (ZSI/WGRC/V/A/966) of *Mysticellus franki* gen. et sp. nov. in preservation. (**a**) Dorsal view; (**b**) ventral view; (**c**) schematic illustration of lateral colour pattern; (**d**) lateral view of head; (**e**) ventral view of hand; (**f**) ventral view of foot.

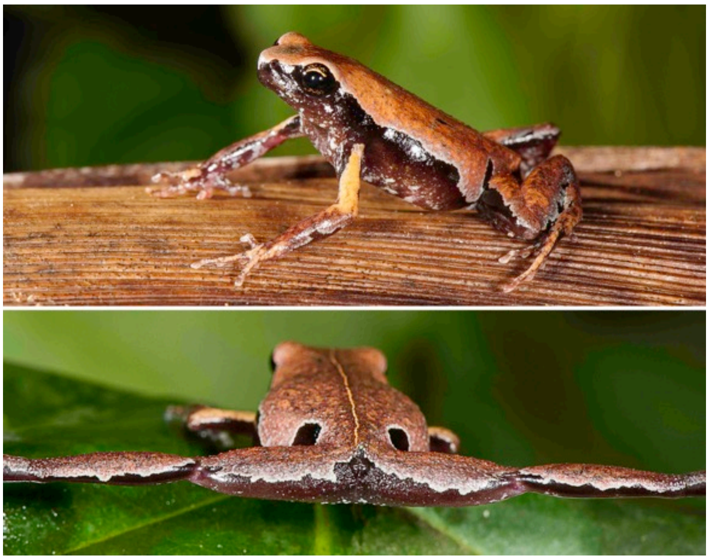

Supplementary Figure S2. Paratype specimen (ZSI/WGRC/V/A/970) of *Mysticellus franki* gen. et sp. nov. in life. (**a**) Dorsolateral view; (**b**) posterior region of back showing two 'false-eye' like spots and thigh markings.

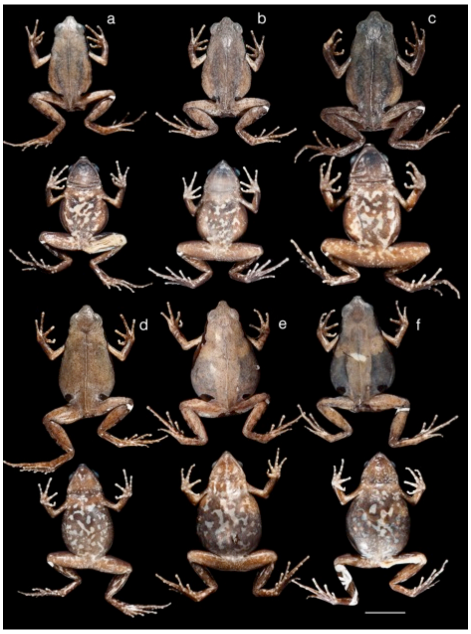

Supplementary Figure S3. Dorsal (above) and ventral (below) view of paratype specimens of *Mysticellus franki* gen. et sp. nov. in preservation.
(**a**) ZSI/WGRC/V/A/967 (male);
(**b**) ZSI/WGRC/V/A/970 (male);
(**c**) ZSI/WGRC/V/A/968 (male);
(**d**) ZSI/WGRC/V/A/969 (male);
(**e**) ZSI/WGRC/V/A/972 (female);
(**f**) ZSI/WGRC/V/A/971 (female).

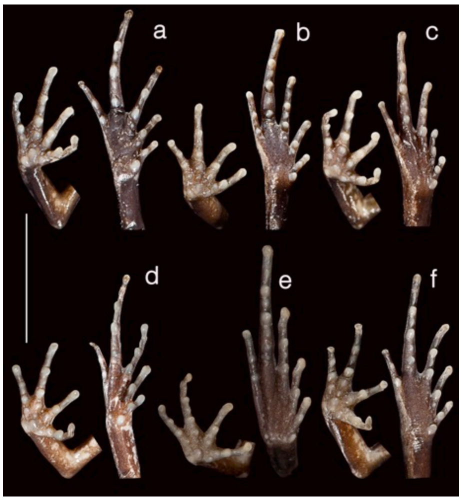

Supplementary Figure S4. Hand (left) and foot (right) of paratype specimens of *Mysticellus franki* gen. et sp. nov. in preservation.
(**a**) ZSI/WGRC/V/A/967 (male);
(**b**) ZSI/WGRC/V/A/970 (male);
(**c**) ZSI/WGRC/V/A/968 (male);
(**d**) ZSI/WGRC/V/A/969 (male);
(**e**) ZSI/WGRC/V/A/972 (female);
(**f**) ZSI/WGRC/V/A/971 (female).

Hand musculature. *Mysticellus franki* **gen. et sp. nov.** differs from other members of the family Microhylidae (including genus *Micryletta*) by having the m. flexor teres digiti III ventral to both slips of the m. transversus metacarpus 1 (Burton 1998a; Fig. 1l). Additionally, it has two previously unreported flexor muscles on digits III and IV, one lateral to the m. lumbricalis brevis digiti III and the other medial to the medial slip of the m. lumbricalis brevis digiti IV. Both flexor muscles have a common origin from the distal carpals, just lateral to the m. lumbricalis brevis digiti III. The flexor muscle of digit III inserts on the tegument by a flat tendon (topologically at the medial side of the metacarpal-digital joint). The flexor muscle of the digiti IV converges distally with the medial slip of the m. lumbricalis brevis digiti IV and inserts on the metacarpal-digital joint (Fig. 1m).

The palmar hand musculature of *Mysticellus franki* **gen. et sp. nov.** and *Micryletta* is shown in Figures 1l–n. Specimens examined: *Mysticellus franki* **gen. et sp. nov.**, SDBDU 2015.2865 and SDBDU 2015.2870; *Micryletta inornata*, KU 328192, from Thailand. Removed elements in Figure 1l: ADD V, APAL, CP III, TS II, FT II, LBB II–V, LBL IV–V, PAL, PML, TS II–V, and muscles 1–2. Removed elements in Figure 1m: CP III, LBL IV–V, TS II–V. Removed elements in Figure 1n: CP III, FT II, LBB II–V, LBL IV–V, TS II–V.

Description of tadpole (Gosner stage 34). A sinistral spiracle is present and takes the shape of a short tube. Spiracular opening is located above the ventral tail musculature margin. Tubular vent tube is seen ventrally and the aperture of the vent tube opening is located

medially in line with the plane of the ventral fin. Tail is composed of bilateral myotomic muscle masses divided by V-shaped septa and has unequal tail membranes on either side of the tail musculature; tail is tapering at its distal end and ends in a whip-like flagellum. Tail musculature does not extend towards the tip of the tail and ends halfway; a weak trail of muscles extends up to the tail tip. Dorsal fins originate near the tail-body junction. Margin of the lower fin is not parallel to the margin of the tail muscle. Body is bulbous-shaped dorsally with a narrower and shorter anterior region, and a wider and longer posterior region. Snout is rounded laterally (from the eyes to the base of the upper labium) and dorsally. Eyes are small, bulbous-shaped and positioned laterally. Pigmentation is visible around the narial depressions, and the narial depressions are located much closer to the eyes than the snout. Mouth appears as a slit opening and the oral disc is absent. No jaw sheaths and papillae are seen and the mouth lacks keratodonts (Supplementary Fig. 1g–i).

Measurements (in mm) are as follows: Total length (TL) = 28.78, snout to vent length (SVL) = 8.98, distance from tip of snout to insertion of upper tail fin (SU) = 7.34, distance from vent to tip of tail (VT) = 19.80, maximum height of body (BH) = 5.32, maximum width of body (BW) = 6.51, maximum height of tail (HT) = 6.30, tail muscle height (TMH) = 5.98, tail muscle width (TMW) =1.96, internarial distance (NN) = 1.24, and interpupular distance (PP) = 5.36.

Measurements and abbreviations for adult morphometry. The following measurements were taken: SVL (snout–vent length), HW (head width, at the angle of the jaws), HL (head length, from rear of mandible to tip of snout), SL (snout length, from tip of snout to anterior orbital border), MN (distance from the rear of the mandible to the nostril), EL (eye length, horizontal distance between bony orbital borders), EN (distance from the front of the eye to the nostril), NS (distance from the nostril to the tip of the snout), IN (internarial distance), IFE (internal front of the eyes, shortest distance between the anterior orbital borders), IBE (internal back of the eyes, shortest distance between the posterior orbital borders), IUE (inter upper eyelid width, the shortest distance between the upper eyelids), UEW (maximum upper eyelid width), TYD (maximum tympanum diameter), TYE (tympanum to eye distance), FAL (forearm length, from flexed elbow to base of outer palmar tubercle), HAL (hand length, from base of outer palmar tubercle to tip of third finger), OPTL (length of outer palmar tubercle), IPTL (length of inner palmar tubercle), PTL (length of center palmar tubercle), FL (finger length), THL (thigh length), SHL (shank length), FOL (foot length, from base of inner metatarsal tubercle to tip of fourth toe), TFOL (distance from the heel to the tip of the fourth toe), OMTL (length of outer metatarsal tubercle), IMTL (length of inner metatarsal tubercle), TL (toe length). Digit number is represented by roman numerals I–V.

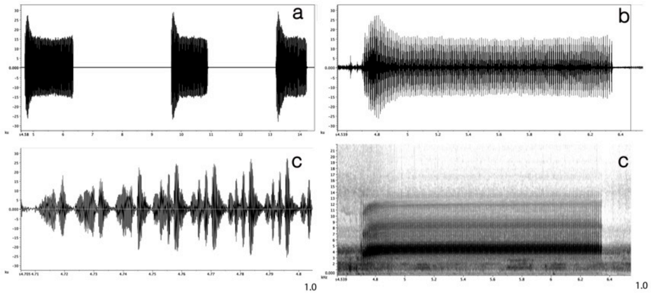

Supplementary Figure S5. Call structure of male *Mysticellus franki* gen. et sp. nov. (ZSI/WGRC/V/A/966). (**a**) 14.5 second segment showing three calls; (**b**) single call; (**c**) one second segment; (**d**) spectrogram of one second segment**.**

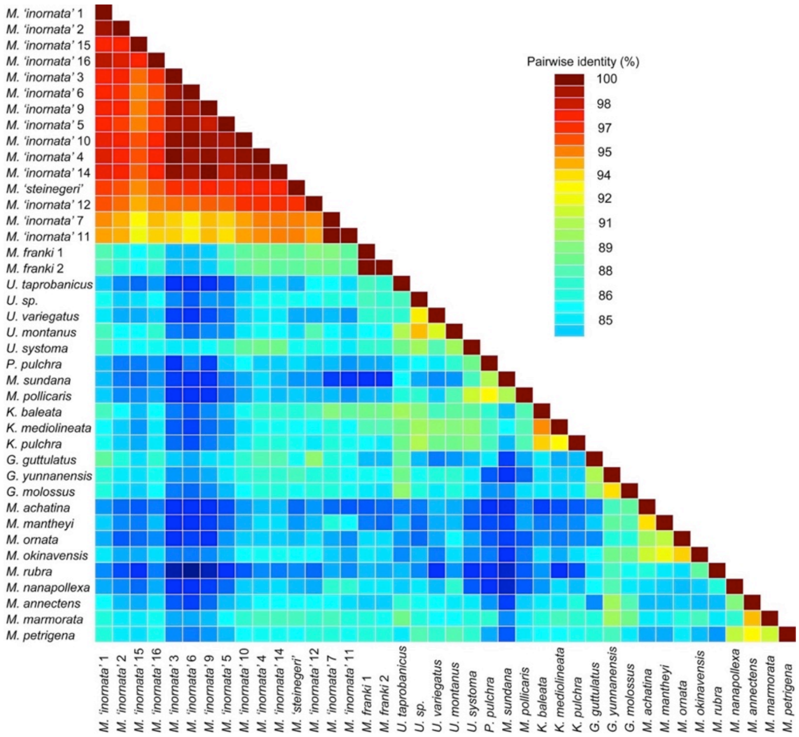

Supplementary Figure S6. Pairwise identity matrix for the mitochondrial 16S rRNA gene sequences representing seven previously recognized genera in the subfamily Microhylinae and *Mysticellus franki* gen. et sp. nov.

Supplementary Table S1. List of DNA sequences used in the study. Reference for sequences retrieved from NCBI GenBank are indicated in superscript. Type species of microhyline genera are indicated with the asterisk symbol. Multiple Genbank sequences are included for *Micryletta inornata*.

Species	Collection locality	Voucher no.	Accession numbers (16S, COI, Tyr, BDNF, His3, SIA1)
**Microhylinae**			
Glyphoglossus guttulatus	Vietnam: Gia-Lai Province, Ankhe district	FMNH 252955	DQ283144[1], KM509768[2], DQ282937[1], KM509233[2], DQ284228[1], DQ282700[1]
Glyphoglossus yunnanensis	China: Yunan province Bao Shan Pref	CAS 242184	KM509102[2], KM509769[2], KM509902[2], KM509234[2], KM509506[2], KM509637[2]
*Glyphoglossus molossus**	Myanmar: Sagaing	CAS 210056	KM509135[2], KM509798[2], KM509928[2], KM509269[2], KM509540[2], KM509671[2]
Kaloula baleata	Indonesia: Lesser Sunda: Sumba	KUHE 32313	KM509153[2], KM509817[2], KM509944[2], KM509289[2], KM509558[2], KM509689[2]
Kaloula mediolineata	Thailand: Tak, Barrntak	KUHE 35178	KM509155[2], KM509819[2], KM509946[2], KM509291[2], KM509560[2], KM509691[2]
*Kaloula pulchra**	Myanmar: Chattgub WS, Sagaing	USFS 34083	KC180025[5], n.a., KC180314[5], KC180100[5], n.a., n.a.
*Metaphrynella pollicaris**	–	KUZ 21655	AB611934[4], AB611931[4], AB611929[4], AB611930[4], n.a., n.a.
Metaphrynella sundana	Malaysia: Sabah: Lahad Datu district	FMNH 231203	KM509161[2], KM509826[2], KM509952[2], KM509298[2], KM509566[2], KM509697[2]
*Microhyla achatina**	Indonesia: Java: Ungaran	MZB Amp 16402	KM509162[2], KM509827[2], KM509953[2], KM509299[2], KM509567[2], KM509698[2]
Microhyla annectens	–	KUHE 52438	AB611947[4], AB611944[4], AB611942[4], AB611943[4], n.a., n.a.
Microhyla heymonsi	China: Hainan province	MVZ 236751	n.a., EF396055[3], EF395979[3], EF396020[3], n.a., n.a.
Microhyla mantheyi	Malaysia: Selangor: Gombak	KUHE 15726	KM509163[2], KM509828[2], KM509954[2], KM509300[2], KM509568[2], KM509699[2]
Microhyla marmorata	–	KUHE 32455	AB611955[4], AB611952[4], AB611950[4], AB611951[4], n.a., n.a.
Microhyla nanapollexa	Vietnam: Quang Nam Province: Tra My District, Tra Don Commune, Mount Ngoc Linh	AMNH 163686 / AMCC 106460	KM509164[2], KM509829[2], KM509955[2], KM509301[2], KM509569[2], KM509700[2]
Microhyla okinavensis	–	IABHU (living)	AB303950[4], AB303950[4], AB611958[4], AB611959[4], n.a., n.a.
Microhyla ornata	Myanmar: Ma Gawe Reserve, Kalaw, Taunggyi, Sha	CAS230957	KC179995[5], n.a., KC180221[5], KC180087[5], n.a., n.a.
Microhyla petrigena	Malaysia: Sarawak, Bukit Kana	KUHE 53743	KM509165[2], KM509830[2], KM509956[2], KM509302[2], KM509570[2], KM509701[2]
Microhyla pulchra	Vietnam: Vinh Phu Province	MVZ 223797	n.a., EF396056[3], EF395980[3], EF396021[3], n.a., n.a.
Microhyla rubra	Myanmar: Magway Div. Pakoku, Dist.	CAS 215851	KM509166[2], KM509831[2], KM509957[2], KM509303[2], KM509571[2], KM509702[2]
Micryletta '*inornata*' (1)	Laos: Boulapha, Khammouan	FMNH 255121	KC179997[5], n.a., n.a., KC180117[5], n.a., n.a.
Micryletta '*inornata*' (2)	Laos: Khammouan Province, Boualapha district	FMNH:255121	KC822494[8], n.a., n.a., n.a., n.a., n.a.
Micryletta '*inornata*' (3)	Laos: Louangphrabang, Ban Sop Choun	2006.2401	KR827951[9], KR087827[9], n.a., n.a., n.a., n.a.
Micryletta '*inornata*' (4)	Laos: Luangprabang Province, Ban Sop Chuna	K3246	KC180027[5], n.a., KC180272[5], KC180131[5], n.a., n.a.
Micryletta '*inornata*' (5)	Laos: Phongsaly Province, Long Nai Kao	K1956	KC180067[5], n.a., KC180268[5], KC180209[5], n.a., n.a.
Micryletta '*inornata*' (6)	Laos: Phongsali, Long Nai Khao	2005.0179	KR827952[9], KR087828[9], n.a., n.a., n.a., n.a.
Micryletta '*inornata*' (7)	Myanmar: Tanintharyi Div., Kawthaung district	CAS 247206	KM509167[2], KM509832[2], KM509958[2], KM509304[2], KM509572[2], KM509703[2]
Micryletta '*inornata*' (8)	Thailand: Bab Tung Tao, Surat Thani, Bab Na San	AK1090	n.a., n.a., KC180233[5], n.a., n.a., n.a.
Micryletta '*inornata*' (9)	Thailand: Chiang Mai, Doi Chiang Dao	K3068	KR827953[9], KR087829[9], n.a., n.a., n.a., n.a.
Micryletta '*inornata*' (10)	Thailand: Phrae, Mae Yom	KUHE 20497	AB598341[6], n.a., n.a., n.a., n.a., n.a.
Micryletta '*inornata*' (11)	Thailand: Ranong	KUHE 23858	AB634695[6], n.a., n.a., n.a., n.a., n.a.
Micryletta '*inornata*' (12)	–	KUHE 35133	AB611968[4], AB611965[4], AB611964[4], EF396022[4], n.a., n.a.
Micryletta '*inornata*' (13)	–	K3068	n.a., n.a., KC180270[5], KC180114[5], n.a., n.a.
Micryletta '*inornata*' (14)	–	Note: No. X21655	AF215375[7], n.a., n.a., n.a., n.a., n.a.
Micryletta '*inornata*' (15)	–	TZ9892	AF285206[10], n.a., n.a., n.a., n.a., n.a.
Micryletta '*inornata*' (16)	–	TZ98110	AF285207[10], n.a., n.a., n.a., n.a., n.a.
Micryletta '*steinegeri*'	Taiwan: Yunlin	KUHE 35937	AB634696[6], n.a., n.a., n.a., n.a., n.a.

Mysticellus franki (1)	India: Kerala, Wayanad, Suganthagiri	ZSI/WGRC/V/A/967	MK285340, MK285341, MK285345, MK285342, MK285343, MK285344
Mysticellus franki (2)	India: Kerala, Wayanad, Suganthagiri	SDBDU 2014.2599	MK285346, MK285347, MK285351, MK285348, MK285349, MK285350
*Phrynella pulchra**	–	UKM HC 820	AB611976[4], AB611973[4], AB611971[4], AB611972[4], n.a., n.a.
Uperodon mormorata	–	Not preserved	AB612000[4], AB611997[4], AB611995[4], AB611996[4], n.a., n.a.
Uperodon 'obscurus'	Sri Lanka: Kandy	MNHN 2000.628	n.a., EF396065[3], EF395989[3], EF396030[3], n.a., n.a.
Uperodon anamalaiensis	India: Kerala, Methooty stream, Idukki district	SDBDU 1342	KM509186[2], KM509854[2], KM509978[2], KM509326[2], KM509590[2], KM509722[2]
*Uperodon systoma**	India: Orissa	SDB 1704	KM509209[2], KM509878[2], KM510000[2], KM509350[2], KM509613[2], KM509744[2]
Uperodon taprobanicus	Sri Lanka: Kurunegala district, Karawaddana	SDB 1151	KM509154[2], KM509818[2], KM509945[2], KM509290[2], KM509559[2], KM509690[2]
Uperodon variegatus	India: Kerala, Palakkad	SDBDU 1354	KM509187[2], KM509855[2], KM509979[2], KM509327[2], KM509591[2], KM509723[2]
**Asterophryinae**			
Asterophrys turpicola	Indonesia: West Papua: Raja Ampat	ABTC90180	KM509095[2], KM509761[2], KM509895[2], KM509226[2], KM509499[2], KM509630[2]
Cophixalus balbus	Papua New Guinea: Sandaun Province: Utai	ABTC 111930	KM509115[2], KM509780[2], KM509911[2], KM509248[2], KM509519[2], KM509650[2]
Gastrophrynoides immaculatus	Peninsular Malaysia: Negeri Sembilan: Gunung Besar Hantu	UKM HC 279	AB611908[4], AB611905[4], AB611903[4], AB611904[4], KM509538[2], KM509669[2]
**Dyscophinae**			
Dyscophus guineti	Pet trade (genus is endemic to Madagascar)	AMNH	KM509127[2], KM509790[2], KM509922[2], KM509260[2], KM509531[2], KM509662[2]
Dyscophus insularis	Madagascar	AMNH-A 173883	KM509128[2], KM509791[2], KM509923[2], KM509261[2], KM509532[2], KM509663[2]

Supplementary Table S2. Uncorrected intraspecific genetic distances within eight recognized genera in subfamily Microhylinae for the mitochondrial 16S rRNA gene sequences. N represents number of samples. The original p-distances are shown in percent.

Species	Mean	Min	Max	N
Glyphoglossus	7.4	5.7	8.7	3
Kaloula	5.3	4.3	6.0	3
Metaphrynella	7.5	–	–	2
Microhyla	9.6	4.6	12.1	9
Micryletta	2.6	0	5.8	15
Mysticellus	0	–	–	2
Phrynella	–	–	–	1
Uperodon	7.8	5.1	9.6	5

Supplementary Table S3. Uncorrected interspecific genetic distances between eight recognized genera in subfamily Microhylinae for the mitochondrial 16S rRNA gene sequences. Mean and range values (in parentheses) are provided. The original p-distances are shown in percent.

Genus	*Glyphoglossus*	*Kaloula*	*Metaphrynella*	*Microhyla*	*Micryletta*	*Phrynella*	*Uperodon*	*Mysticellus*
Glyphoglossus	–							
Kaloula	10.9 (10.5–11.8)	–						
Metaphrynella	13.3 (11.5–15.3)	10.7 (9.2–12.4)	–					
Microhyla	9.9 (7.9–12.4)	11.5 (9.2–14.1)	13.3 (10.5–17.0)	–				
Micryletta	10.7 (9.4–13.5)	10.6 (8.2–13.2)	12.5 (11.1–14.6)	11.7 (9.0–16.2)	–			
Phrynella	12.0 (11.1–12.5)	10.5 (10.0–11.1)	6.7 (6.0–7.3)	13.0 (11.1–14.6)	12.2 (11.0–14.4)	–		
Uperodon	10.5 (8.9–12.0)	8.6 (7.7–9.4)	10.5 (7.0–12.6)	11.7 (9.4–14.0)	11.6 (10.0–14.4)	10.1 (8.5–10.7)	–	
Mysticellus	11.0 (10.2–12.0)	9.2 (8.4–10.5)	12.8 (11.3–14.3)	11.3 (9.6–13.2)	9.3 (8.2–11.3)	11.5 (11.5–11.6)	9.9 (9.2–10.4)	–

Supplementary Table S4. Substitution models inferred in PartitionFinder 2 for gene partitions used for divergence time analysis.

Gene fragment	# sites	Best Model
tyrosinase (Try)	534	K80+G
brain-derived neurotrophic factor (BDNF)	701	K80+I+G
histone H3 (His3)	330	HKY+G+X
seven in absentia homolog 1 (SIA1)	399	HKY+G+X
cytochrome oxidase I (COI)	660	HKY+I+G+X
ribosomal subunit 16S rRNA (16S)	464	SYM+I+G

References

1. Frost, D.R. *et al*. The amphibian tree of life. *Bull. Am. Mus. Nat. Hist*. **297**, 1–370 (2006).
2. Peloso, P. L. *et al*. The impact of anchored phylogenomics and taxon sampling on phylogenetic inference in narrow-mouthed frogs (Anura, Microhylidae). *Cladistics*. **32(2)**, 113–140 (2016).
3. Van der Meijden, A. *et al*. Nuclear gene phylogeny of narrow-mouthed toads (Family: Microhylidae) and a discussion of competing hypotheses concerning their biogeographical origins. *Mol. Phylogenet. Evol*. **44(3)**, 1017–1030 (2007).
4. Kurabayashi, A. *et al*. From Antarctica or Asia? New colonization scenario for Australian-New Guinean narrow mouth toads suggested from the findings on a mysterious genus Gastrophrynoides. *BMC Evol. Biol*. **11(1)**, 175 (2012).
5. de Sá, R. O. *et al*. Molecular phylogeny of microhylid frogs (Anura: Microhylidae) with emphasis on relationships among New World genera. *BMC Evol. Biol*. **12**, 241 (2012).
6. Matsui, M. *et al*. Systematic relationships of Oriental tiny frogs of the family Microhylidae (Amphibia, Anura) as revealed by mtDNA genealogy. *Mol. Phylogenet. Evol*. **61(1)**, 167–176 (2011).
7. Vences, M. Phylogenetic studies of ranoid frogs (Amphibia:Anura), with a discussion of the origin and evolution of the vertebrate clades of Madagascar (Bonn, 2000).
8. Blackburn, D.C. *et al*. An adaptive radiation of frogs in a Southeast Asian island archipelago. *Evolution*. **67(9)**, 2631–2646 (2013).
9. Grosjean, S., Ohler, A., Chuaynkern, Y., Cruaud, C. & Hassanin, A. Improving biodiversity assessment of anuran amphibians using DNA barcoding of tadpoles. Case studies from Southeast Asia. *C. R. Biol*. **338(5)**, 351–361 (2015).
10. Ziegler, T. Research on the herpetofauna in a wetland preserve in the south of North Vietnam (Bonn, 2000).

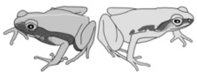

Supplement: Supplementary file 1 — Electronic Supplementary Material [file 41598_2018_38133_MOESM1_ESM.pdf]
